# Successful treatment with joint retention of *Mycobacterium ulcerans* prosthetic joint infection in a patient with rheumatoid arthritis

**DOI:** 10.1371/journal.pntd.0013887

**Published:** 2025-12-26

**Authors:** Madeleine Bangham, Eloise Williams, Derek Neoh, Janine Trevillyan, Nicola Sexton-Oates, Paul D. R. Johnson

**Affiliations:** 1 Infectious Diseases and Immunology Department, Austin Hospital, Heidelberg, Victoria, Australia; 2 Department of Plastic and Reconstructive Surgery, Austin Hospital, Heidelberg, Victoria, Australia; 3 Department of Infectious Diseases, University of Melbourne at the Peter Doherty Institute of Infection and Immunity, Melbourne, Victoria, Australia; University of Surrey Faculty of Health and Medical Sciences, UNITED KINGDOM OF GREAT BRITAIN AND NORTHERN IRELAND

## Abstract

*Mycobacterium ulcerans*, the causative agent of Buruli ulcer, is an emerging pathogen in southeastern Australia. Disease typically presents as a single cutaneous lesion although atypical and multi-focal infection does occur, and in these cases a lack of clinical suspicion may delay the diagnosis. With increased exposure of older and medically immunosuppressed populations to *M. ulcerans* transmission, we need a clearer understanding of how age and underlying immune dysfunction may alter both the risk of acquisition and the clinical presentation of the infection. We present the first known case of *M. ulcerans* infection involving a prosthetic joint. A 68-year-old female with rheumatoid arthritis immunosuppressed with methotrexate and prednisolone presented with an acutely painful, erythematous prosthetic metacarpophalangeal joint. She also reported multiple cutaneous ulcers, developing over the preceding year. Laboratory investigations revealed raised inflammatory markers, and a complex peri-prosthetic collection was seen on imaging. Swabs from the cutaneous ulcers and joint washout for *M. ulcerans* PCR and culture positive. PET-CT demonstrated the presence of further sub-clinical cutaneous lesions. The infection was successfully managed with a surgical washout, implant retention and 12 weeks of oral antimicrobials. This case highlights an atypical presentation of Buruli ulcer in an immunosuppressed patient, and the management considerations involved. The broadening geographical distribution of *M. ulcerans* will place a growing population of immunosuppressed patients at risk; thus awareness amongst clinicians is crucial to ensure prompt diagnosis and treatment.

## Author summary

*Mycobacterium ulcerans* is an environmental pathogen which causes Buruli ulcer. In southeastern Australia it is transmitted by mosquitoes from a possum reservoir, and the geographical areas where it occurs are expanding. It usually causes single, painless skin ulcers, but in immunosuppressed people, Buruli ulcer can be multi-focal and affect deeper anatomical structures. This case describes a patient with rheumatoid arthritis treated with immunosuppressing medication, who developed disseminated Buruli ulcer affecting her skin and a prosthetic joint in her hand. The infection was successfully treated with surgery to wash out the joint, retention of the implant, and 12 weeks of antibiotics. It is important to consider Buruli ulcer as a diagnosis in immunosuppressed patients who live in or visit Buruli ulcer endemic regions, who present with unusual and persisting infections of their skin, bones or joints. Presentations in this group of patients may be unusual and diagnosis requires targetted testing that won’t be performed unless specifically requested. Clinicians who practice in Buruli-endemic areas must be aware of this important infection and ensure appropriate investigations are performed to enable directed treatment of *M. ulcerans*.

## Introduction

Buruli ulcer is a necrotising skin and soft tissue infection caused by *Mycobacterium ulcerans* [1]. Buruli ulcer is a geographically restricted infection but the incidence in southeastern Australia has increased exponentially over the past two decades, with the establishment of multiple new endemic areas [2]. The environmental reservoir in this region is the native possum [3,4] with transmission to humans via mosquitoes [4,5].

Disease typically presents as a single, usually painless and slowly enlarging lesion on a limb, but can occur anywhere [6]. Occasionally, Buruli ulcer can cause osteomyelitis although this has been rare in Australia [7,8]. In a recent review of 1750 consecutively notified cases from the southeastern state of Victoria, only 2.3% were multi-focal [2]. Multi-focal disease has been associated with older age (>60 years) in Australia [9], and with Human Immunodeficiency Virus (HIV) infection in Africa [10].

Despite the rising incidence, little is yet known about how immunosuppression from comorbidities or immunosuppressive medications may alter risk or severity of Buruli ulcer. We present the first reported case of multi-focal *M. ulcerans* infection with prosthetic joint involvement in a patient with rheumatoid arthritis on methotrexate and prednisolone. This case highlights the need for increased clinical suspicion of Buruli ulcer in immunosuppressed individuals, particularly those presenting with skin, soft-tissue, bone and joint infections in endemic regions.

### Case

A 68-year-old female presented with a two-day history of atraumatic pain, swelling and erythema in her right fifth metacarpophalangeal (MCP) joint, where she had a silicone prosthesis. She had a limited range of movement in the joint, but denied fevers or other systemic features of infection. She also reported six non-healing cutaneous lesions which had developed sequentially over the past 12 months, on her bilateral elbows, right knee and left thigh. These were erythematous and mildly tender to palpation, and had been diagnosed as rheumatoid nodules by her General Practitioner. She had received three courses of oral flucloxacillin and cephalexin with no effect. Two months prior, she had had an incision and drainage of one of the lesions on her thigh, which had failed to resolve. Swabs of the lesions were sent for routine Gram stain, culture and sensitivity but were reported as negative.

Her medical history included a diagnosis of rheumatoid arthritis, managed with 20 mg methotrexate weekly and 2 mg prednisolone daily. She had significant rheumatoid hand deformities, so had undergone bilateral second to fifth MCP silicone joint replacements to maintain dexterity as a pianist. She also had hypothyroidism for which she took 100 μg levothyroxine daily, and well-controlled bipolar affective disorder for which she took risperidone 0.5 mg daily, lithium 375 mg daily and sertraline 100 mg daily. She had lived for many years in metropolitan Melbourne outside any currently declared Buruli ulcer endemic area [11]. However, she regularly travelled to a town on the Bellarine Peninsula, 100 km by road from Melbourne, where transmission of *M. ulcerans* has been increasingly reported after first appearing there in 1998 [12]. She had recently travelled overseas to New Zealand, but had no other relevant occupational or environmental exposure history.

On examination, the prosthetic right fifth MCP joint was hot, swollen and exquisitely tender to palpation, with a small overlying area of ulceration and purulent exudate. The larger lesions had undermined edges. Further examination confirmed the other cutaneous lesions on her limbs, which were slightly raised, tender and with a central area of ulceration. All vital signs were within normal limits.

Blood tests revealed a mildly elevated white cell count of 12.4 x 10^9^/L (4.0-12.0), with neutrophils of 8.6 x 10^9^/L (2.0-8.0). C-reactive protein was 87.0 mg/L (<5.0). Renal function, liver function and electrolytes were normal. An X-ray of the hand showed no osteomyelitis, and an ultrasound scan of the joint demonstrated a complex heterogenous fluid collection around her prosthetic fifth MCP joint, extending in to the extensor tendons. Standard bacterial blood cultures were negative. Swabs from all the ulcers were sent for standard bacterial culture, herpes multiplex polymerase chain reaction (PCR), mycobacterial culture and *M. ulcerans* PCR.

**Fig 1:**
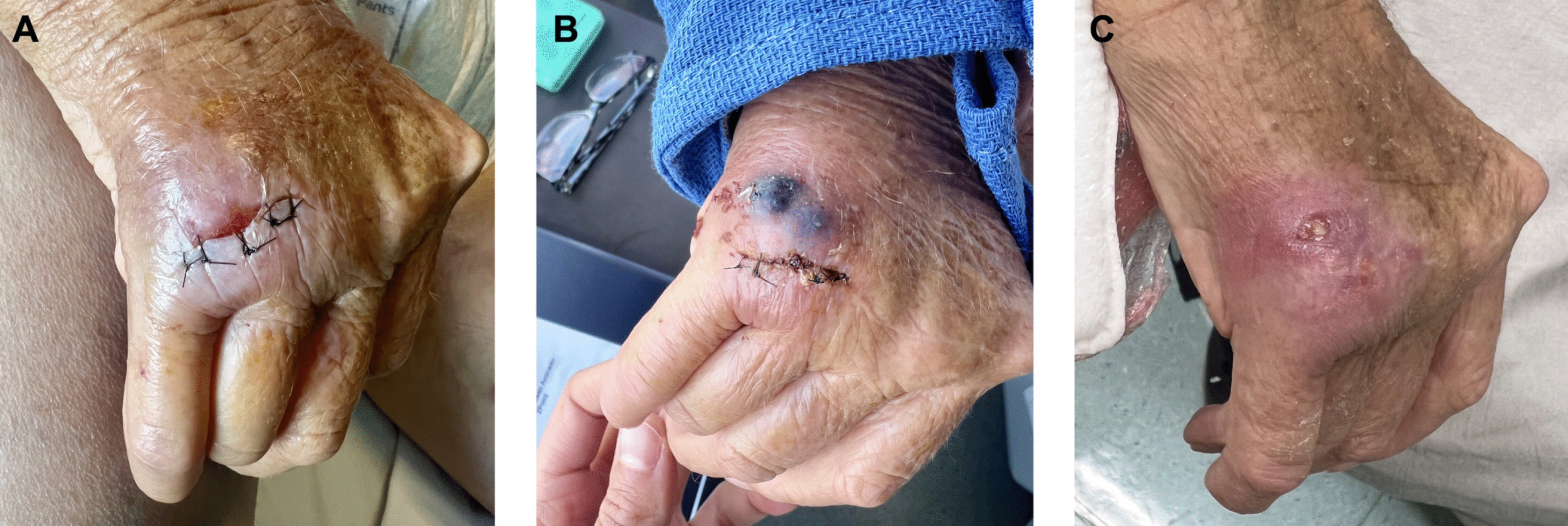
Prosthetic right fifth metacarpophalangeal joint infection with *Mycobacterium ulcerans.* A: immediately after joint washout. B: development of paradoxical reaction. **C**: following completion of 12 weeks of antibiotics.

The prosthetic joint and extensor tendon compartment was surgically washed out by the plastic surgery team (Fig 1A). Intraoperative findings revealed purulent material surrounding the joint and multiple extensor tendons. The prosthetic joint was retained. Swabs and tissue taken intraoperatively were sent for bacterial and mycobacterial culture, and *M. ulcerans* PCR.

Swabs from the cutaneous lesions and joint washout were PCR-positive and culture positive for *M. ulcerans*, and no other bacterial pathogens were isolated. A diagnosis of multi-focal *M. ulcerans* infection with an associated prosthetic joint infection was made.

**Fig 2:**
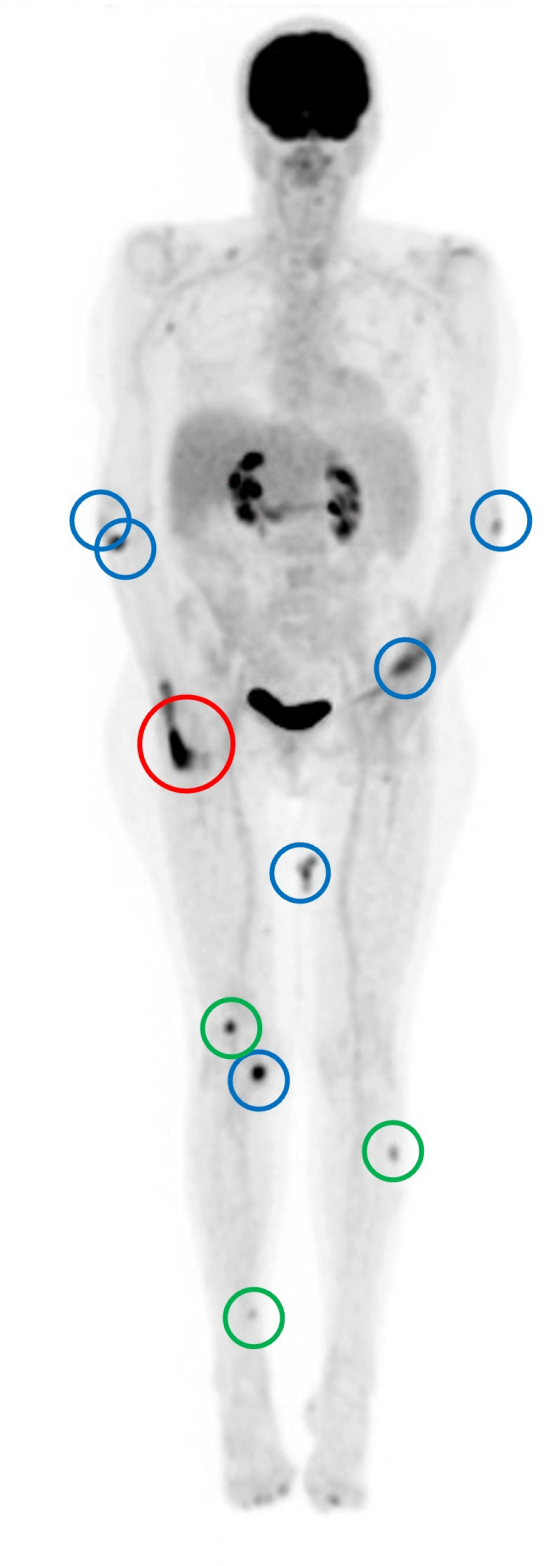
PET-CT scan demonstrating PET-avidity in the prosthetic right fifth metacarpophalangeal joint, and nine other cutaneous sites of infection. The infected joint is marked in red (*Mycobacterium ulcerans* PCR-positive). The initial six cutaneous lesions are marked in blue (*Mycobacterium ulcerans* PCR-positive). The three subclinical lesions are marked in green.

A whole-body PET-CT scan (Fig 2) was performed to rule out involvement of internal organs, which demonstrated PET-avidity of the infected prosthetic joint and six known cutaneous lesions. It also revealed a further three cutaneous sites of sub-clinical disease, which were minimally visible on examination and that the patient had not noticed herself.

Following surgery, she was commenced on oral rifampicin 600 mg daily and oral ciprofloxacin 500 mg twice daily [13]. Ciprofloxacin was chosen over clarithromycin to avoid drug interactions with her long standing psychotropic medications. Methotrexate and prednisolone were withheld, and under the guidance of the rheumatology team hydroxychloroquine 200mg daily was commenced. After one week of antimicrobial therapy, she experienced worsening pain and swelling overlying the affected joint (Fig 1B). The three previously asymptomatic sites identified on PET-CT became clinically apparent, with evolving erythema and ulceration. She was therefore commenced on 50 mg prednisolone to treat a multi-focal paradoxical reaction [13], which was weaned over the following four weeks. She completed a total of 12 weeks of treatment, with resolution of her prosthetic joint infection and multi-focal cutaneous lesions (Fig 1C). To date, six months after initial presentation, she has had no recurrence of disease and has returned to playing the piano. She remains on hydroxychloroquine 200 mg daily and prednisolone 2 mg daily for her rheumatoid arthritis.

## Discussion

To our knowledge, this is the first reported case of *M. ulcerans* infection involving a prosthetic joint. The infected MCP joint prosthesis was part of an extensive multi-focal and deep-seated infection, in the context of immunosuppression from rheumatoid arthritis that was being managed with methotrexate and prednisolone.

Buruli ulcer, caused by the environmental pathogen *M. ulcerans*, affects both immunocompetent and immunocompromised individuals. It has been extensively studied in immumocompetent populations but much less is known about the disease in patients taking medical immunosuppression.

The pathogenesis of *M. ulcerans* infection is explained by the production of the unique, plasmid-encoded [14] diffusible toxin mycolactone [15]. Mycolactone causes concentration-dependent apoptosis and necrosis, and alters the function of a range of innate and adaptive immune cells [16]. These broad effects are mediated by its binding to and inhibition of the Sec61 complex, a heterotrimeric protein channel in the endoplasmic reticulum (ER) of eukaryotic cells [17]. Sec61 functions as a porin that facilitates export of ribosomally produced proteins destined to be secreted or bound to and presented on the external cell membrane [18]. Both innate and adaptive immune cells rely on cytokine, chemokine and receptor production for their normal function, all of which are interfered with by Sec61 blockade [19].

Buruli ulcer progresses when intense toxin-mediated local immunosuppression allows bacterial multiplication to continue unimpeded. Nevertheless, the status of the host’s immune function also affects the clinical manifestations of their disease. Patients with Buruli ulcer and HIV/AIDS co-infection are more likely to have severe Buruli ulcer indicating that in normal hosts, the systemic immune response continues to maintain a degree of control despite the profound local immunosuppression [10]. The important role of systemic host immunity is well illustrated in our case where medical immunosuppression for rheumatoid arthritis altered the clinical features of Buruli ulcer, leading to deep infection at multiple sites. A recent similar local case of multi-focal disease has been reported in a solid organ transplant patient [20] and in an Australian cohort study, immunosuppressive medications such as corticosteroids were associated with an increased rate of antimicrobial treatment failure [21]. A case-control study from southeastern Australia reported a trend towards Buruli ulcer being more common in patients with immunocompromising conditions although this did not reach statistical significance [21].

Whilst *M.ulcerans* infection can occur at any age, notifications in Victoria, Australia are highest in those aged over 60 years old [22]. Prevalence of prosthetic joint replacements and diseases requiring medical immunosuppression also increase with age; therefore extension of *M.ulcerans* endemic areas could expose a greater population of aging patients to risk of prosthetic joint involvement or multifocal infection such as in the case described.

Our patient’s experience of a significant lag between symptom onset and microbiological diagnosis is a frequent occurrence when patients present to doctors unfamiliar with Buruli ulcer [23]. *M. ulcerans* specific PCR testing is rapid and sensitive [24,25] but requires clinical suspicion. The incubation period of Buruli ulcer is long, with a median of 4.5-4.8 months [26,27]. Transmission occurs in the mosquito season in the warmer months (November to April) [28] but patients typically first present for care in winter and may not recall their exposure many months previously. Notably, our patient’s contact with a known endemic area was not appreciated initially and was only revealed on direct questioning, leading to our decision to test for *M. ulcerans*. Clinicians need to be aware of the changing epidemiology of Buruli ulcer in southeastern Australia, and maintain a low threshold for testing by *M. ulcerans*-specific PCR, as standard non-targeted microbial swabs are not diagnostic and may be misleading. Given the rising incidence of Buruli ulcer in densely populated urban areas [11], the number of immunosuppressed individuals exposed to *M. ulcerans* will grow.

There is strong evidence to support short treatment courses of eight weeks with a combination of rifampicin and clarithromycin or fluoroquinolones for *M. ulcerans* [13]. However, given the patient’s underlying immunocompromise (a risk factor for treatment failure [21]) and the involvement of a prosthetic joint, a decision was made to extend this to 12 weeks [29]. Management of mycobacterial prosthetic joint infections is incompletely defined. A strategy of surgical washout with implant retention followed by prolonged antimicrobial therapy was selected based on inferences drawn from *Mycobacterium tuberculosis* prosthetic joint infections [30,31]. Close follow-up after completion of antimicrobials to assess for relapse is imperative.

## Conclusion

This case is the first reported instance of *M. ulcerans* prosthetic joint infection. It highlights that patients immunosuppressed from underlying disease and medication, particularly those with impaired IFN-y/Th1 immune function, may be at risk of multi-focal or atypical sites of infection.

Prosthetic joint infection due to *M. ulcerans* can be successfully managed with surgical washout, implant retention, and combination antimicrobial therapy**.** Caution must be taken when withholding immunosuppressive medications, as immune reconstitution may enhance paradoxical reactions. As endemic zones expand, human populations age and rates of medical immunosuppression increase, awareness of atypical Buruli presentations will be increasingly important for timely diagnosis and treatment.

### Ethics statement

No Human Research Ethics Committee review was required for this report, as it was considered a retrospective review of an individual case.

### Consent

The case-patient described in this manuscript has given written informed consent (as outlined in the PLOS consent form) to publication of their case details. The patient has reviewed the final manuscript. All reasonable efforts were made to protect their anonymity by removing any identifying details.
